# Volumetric Humeral Canal Fill Ratio Effects Primary Stability and Cortical Bone Loading in Short and Standard Stem Reverse Shoulder Arthroplasty: A Biomechanical and Computational Study

**DOI:** 10.3390/jimaging10120334

**Published:** 2024-12-23

**Authors:** Daniel Ritter, Patric Raiss, Patrick J. Denard, Brian C. Werner, Peter E. Müller, Matthias Woiczinski, Coen A. Wijdicks, Samuel Bachmaier

**Affiliations:** 1 Department of Orthopedic Research, Arthrex, 81249 Munich, Germany; 2 Department of Orthopaedics and Trauma Surgery, Musculoskeletal University Center Munich (MUM), University Hospital, LMU Munich, 80336 Munich, Germany; 3 OCM Clinic, 81369 Munich, Germany; 4 Southern Oregon Orthopedics, Medford, OR 97504, USA; 5 Department of Orthopaedic Surgery, University of Virginia Health System, Charlottesville, VA 22908, USA; 6 Experimental Orthopaedics University Hospital Jena, Campus Eisenberg, Friedrich-Schiller-University, 07607 Eisenberg, Germany

**Keywords:** reverse shoulder arthroplasty, short stem, standard stem, CT imaging, canal fill, stress shielding, micromotion, bone deformation, biomechanics

## Abstract

Objective: This study evaluated the effect of three-dimensional (3D) volumetric humeral canal fill ratios (VFR) of reverse shoulder arthroplasty (RSA) short and standard stems on biomechanical stability and bone deformations in the proximal humerus. Methods: Forty cadaveric shoulder specimens were analyzed in a clinical computed tomography (CT) scanner allowing for segmentation of the humeral canal to calculate volumetric measures which were verified postoperatively with plain radiographs. Virtual implant positioning allowed for group assignment (VFR < 0.72): Standard stem with low (*n* = 10) and high (*n* = 10) filling ratios, a short stem with low (*n* = 10) and high filling ratios (*n* = 10). Biomechanical testing included cyclic loading of the native bone and the implanted humeral component. Optical recording allowed for spatial implant tracking and the quantification of cortical bone deformations in the proximal humerus. Results: Planned filling ratios based on 3D volumetric measures had a good-to-excellent correlation (ICC = 0.835; *p* < 0.001) with implanted filling ratios. Lower canal fill ratios resulted in significantly higher variability between short and standard stems regarding implant tilt (820 N: *p* = 0.030) and subsidence (220 N: *p* = 0.046, 520 N: *p* = 0.007 and 820 N: *p* = 0.005). Higher filling ratios resulted in significantly lower bone deformations in the medial calcar area compared to the native bone, while the bone deformations in lower filling ratios did not differ significantly (*p* > 0.177). Conclusions: Lower canal filling ratios maintain dynamic bone loading in the medial calcar of the humerus similar to the native situation in this biomechanical loading setup. Short stems implanted with a low filling ratio have an increased risk for implant tilt and subsidence compared to high filling ratios or standard stems.

## 1. Introduction

Reverse shoulder arthroplasty (RSA) with stemmed humeral implants has good long-term results with a low humeral loosening rate, but bone resorption rates remain high [1,2,3]. Thus, humeral stems have transitioned to short and stemless designs, accepting the risk of reduced primary fixation stability [4,5,6,7]. Prior clinical studies have shown a correlation between bone resorption and a high canal fill ratio (FR) of humeral implants [8,9,10,11]. Conversely, lower canal fill ratios in short stem RSA are associated with subsidence and varus or valgus malalignment [12]. Initial implant tilt due to reduced mechanical stability was additionally shown to have effects on proximal humeral bone stresses [13]. While thresholds for filling ratios at risk for proximal bone resorption have been defined on plain radiographs (metaphyseal (<0.625) and distal filling ratios (<0.725 and <0.82 depending on metaphyseal ratio)), ref. [14] validation of the distal preoperative measure has shown only moderate predictive accuracy [15]. A three-dimensional (3D) canal fill calculation and its effects on the primary stability and clinical stress shielding may be a valuable tool to increase preoperative planning capabilities.

Related work demonstrated the utility of a volumetric filling ratio (VFR) that was able to predict stress shielding in higher filling ratios more accurately than commonly described two-dimensional filling ratios [16]. Humeral implant primary stability and implant–bone loading patterns vary according to stem type and design and differ from the physiological load transfer patterns in the healthy proximal humerus [13,17]. Finite element analyses (FEA) of stress shielding conditions report a distal load transfer in longer stem lengths, demonstrating the importance of proper stem sizing to achieve a trade-off between adequate primary stability and stress shielding [18,19,20]. Recent assessments of the primary stability of different canal fills leave potential for improvement as only isolated stem stability and 2D imaging were investigated [21]. Segmentation of the humeral canal anatomy in preoperative 3D CT data may help to objectively select the stem size to reduce the risk of implant subsidence and stress shielding [16,22]. Additionally, investigation of the complete humeral implant in relation to the 3D calculated canal filling ratios may improve the understanding of differing implant–bone load transfer patterns [21,23].

The research questions of this study aimed to evaluate the effect of 3D volumetric humeral canal fill ratios of short and standard RSA stems on biomechanical stability. An analysis of the CT-based bone density and the humeral canal in association with the bone micromotion during cyclic testing allowed us to investigate the hypothesis that increased canal fill ratios provide higher primary stability, with less bone loading in the medial proximal humerus.

## 2. Materials and Methods

**Figure 1:**
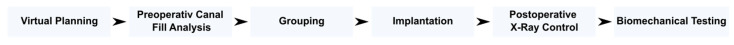
Methodical framework, from virtually planning and developing a volumetric measure of the humeral canal which was used in this study for group assignment and planning of low and high filling ratios. Canal fill ratios were controlled using postoperative X-rays after the implantation and before testing the implanted humeral component biomechanically.

A biomechanical study was performed on 40 cadaveric specimens to evaluate humeral implant stability. Forty cadaveric shoulder specimens (24 male, 16 female; 67 ± 4 years) were procured (Science Care Inc., Phoenix, AZ, USA). None of the specimens showed macroscopic or radiological signs of humeral or glenohumeral pathologies or anomalies. Prior to biomechanical testing, a 3D analysis of the humeral canal was conducted and used to plan the humeral component size (Figure 1).

### 2.1. Virtual Planning

The cadaveric shoulders were scanned with a voxel size of 0.5 mm (120 kVp and 80 mA) in a clinical CT scanner (Siemens SOMATOM Definition AS+, Siemens Healthcare GmbH, Erlangen, Germany) to meet the specifications of a current planning software (Virtual Implant Positioning; Arthrex, Inc., Naples, FL, USA). Patient-specific calibration was performed according to previous studies to make these calculations applicable for multicentric standard preoperative CT data [17,24,25]. Gray scale values were converted into bone mineral density (BMD) values by linearly interpolating grayscale values on defined BMD air fat and muscle values [−840, –80 and 30 mgHA/cm^3^] to reduce intra- and interscanner inaccuracies. Humeral bone density parameters were evaluated to ensure similar bone density distributions in the treatment groups. According to previous studies, the principal bone density parameters (Epiphysis Cylinder BMD (Epi. Cyl. BMD); Epiphysis Cylinder bone volume per total volume (BV/TV), Metaphysis Cylinder BV/TV, Inferior Support (Inf. Sup.) BMD, and age) were evaluated [17,25]. Morphological parameters of each volume of interest were calculated using pixel-counting methods using the respective bone volume (BV) of the total volume (TV) as BV/TV. Bone model development was performed based on CT voxel data imported as a four-dimensional point cloud (i.e., [x, y, z, mgHA/cm^3^]).

### 2.2. Pre- and Postoperative Canal Fill Analysis

**Figure 2:**
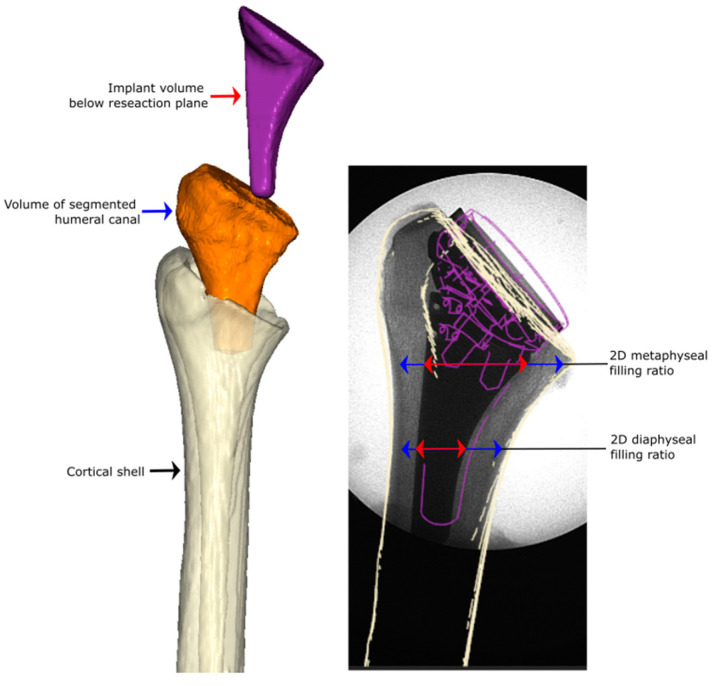
Measurement and calculation of the filling ratios by dividing the red marked measure through the respective blue one. The three-dimensional rendered and segmented CT data on the left side allowed for volumetric calculation of the canal fill ratio (3D VFR). Calculation of the canal fill ratios based on two-dimensional plane radiographs (2D Metaphysis FR and 2D Diaphysis FR) is shown on the right side based on current clinical practice [14,16].

Based on the 3D data, the humeral canal was segmented to calculate volumetric measures after a virtual anatomic humeral head resection according to the surgical technique later performed in the specimen preparation. Standard image processing steps were performed: segmentation of the cortical shell and subtracting it from the whole filled bone resulted in the volumes of interest. The filling ratio calculation in two (2D Metaphysis FR and 2D Diaphysis FR) or three dimensions (3D VFR) was calculated as shown in Figure 2 according to recent clinical studies [14,16]. A commercial CT image processing software (Simpleware ScanIP, Synopsis, Exeter, UK) was used to position the 135° inclined implant virtually with the best-fitting stem and cup size selected (no perforation of the cortical bone). Based on the initial plan, deviating stem and cup sizes were virtually positioned to ensure proper group assignment and implantability for the respective implant types. This resulted in a canal fill ratio range between 0.54 and 0.97.

**Figure 3:**
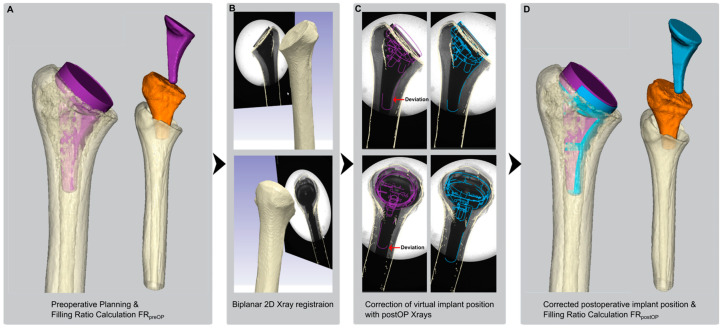
The 2D to 3D registration allowedto validate the accuracy of preoperative canal fill measurements with the actual postoperative implant seating: (**A**). preoperative planning of the humeral implant (purple) and segmentation of the humeral canal (orange), (**B**). registration of postOP X-rays, (**C**). correction of the implant position according to postOP position (blue) and (**D**). calculation of the true postOP canal fill ratio for comparison with the preOP ratio.

An anterior-posterior (AP) and medial-lateral (ML) X-ray was taken before biomechanical testing to validate the preoperatively (preOP) plan with the actual implanted (postOP) position and filling ratio (Figure 3). After preoperative planning and canal fill calculations (Figure 3A), a 3D-2D registration of the humeral bone was performed using preoperative CT data and postoperative X-ray images (Figure 3B). Potential deviations in varus/valgus, rotation, and translation were analyzed (Figure 3C). Postoperative filling ratio calculations were calculated to verify the match between planned and implanted fill (Figure 3D).

### 2.3. Specimen Grouping and Implantation

Following virtual implant selection, a 135° inlay humeral component available in short or standard lengths was implanted (Univers Revers; Arthrex, Inc., Naples, FL, USA). The low versus high filling ratio was defined at a threshold of 0.72, refs. [14,26] resulting in four groups: a standard stem with low (Standard_low_, *n* = 10) and high (Standard_high_, *n* = 10) filling ratios, a short stem with low (SS_low_, *n* = 10), and high filling ratios (SS_high_, *n* = 10).

The cadaveric specimens were stored at −20 °C and thawed at room temperature before tissue preparation and testing. The humeral neck was marked using anatomic landmarks before resecting the humeral head along the anatomic neck perpendicular to the metaphyseal axis using an oscillating saw and a 135° cutting guide. The canal was then prepped according to manufacturer specifications, followed by the placement of the planned humeral component. X-rays were taken to confirm the implant seating. Testing was performed at room temperature, and the tissue was kept moist using saline solution throughout the preparation and testing phases.

### 2.4. Biomechanical Testing

Based on previous biomechanical studies, three levels of load were tested: 220 N, 520 N, and 820 N [24,27,28,29,30,31]. The 220 N load level was applied to mimic 20% body weight (BW) (196 N). The force experienced during rehabilitation arm movements simulates the loading at time-zero after surgery as measured by a telemetric shoulder implant. The 520 N load level was intended to replicate the forces encountered during the initial two months of physical therapy following shoulder arthroplasty, equating to 40% BW (392 N) during resistance training [28]. The highest load level (820 N) simulated peak loads during “normal” use without any weight in hand. As in this rehabilitation phase bone ingrowth already appears, this load level represents a worst-case scenario during rehabilitation. Loads were applied in the coronal plane at a 30° angle from the implant’s central axis, as indicated by in vivo measurements [29,30].

**Figure 4:**
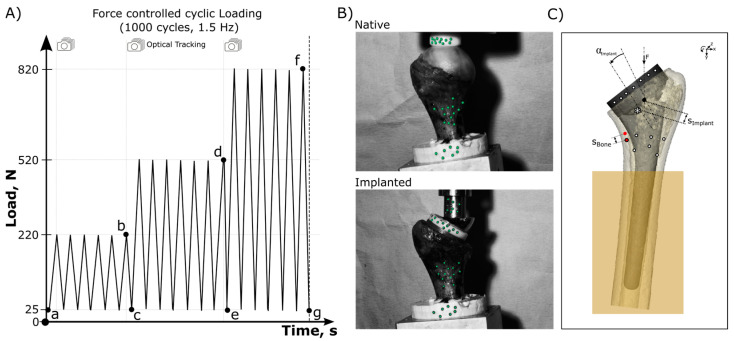
(**A**) Testing protocol shows the loading cycles including the points of data analysis (a–g). (**B**) Experimental cyclic loading setups and the optical tracking points (green) for data analysis. (**C**) Evaluated tracking points during cyclic loading force (F) to analyze implant subsidence and tilt measurements between analysis points a and b, d or f, respectively, (s_imlant_ and α_imlant_, Δab, Δad, and Δaf) at the end of each loading block. Bone micromotion (s_BoneHW_, Δbc, Δde, and Δfg) was evaluated as bone displacement within each final load cycle (hysteresis width (HW)). Total compressive transmission caused deformation of the bone was measured at the end of each loading block (s_BoneTot_, Δab, Δad, and Δaf).

Testing of the native bone was performed before humeral component implantation. The humeral head was cyclically loaded using a custom-made polyethylene stamp that matched the humeral head diameter. After humeral component implantation, the PE of the prosthesis was loaded with the matching glenosphere. Native and humeral component testing was performed in the same setup and specimen orientation to allow for comparison of the two loading situations. In both setups, joint contact pressure was simulated for 1000 cycles per load block in force control mode at a frequency of 1.5 Hz (Figure 4A). A ball bearing was included above the stamp to avoid constraining loads. A single-axis material testing machine (ElectroPuls E3000; Instron, Darmstadt, Germany) was used to apply the loads and investigate micromotion and bone deformation at the steady states within the final cycle of each load block using an optical tracking system (Figure 4B).

Mechanical data were continuously recorded at a sampling rate of 500 Hz. An optical tracking system (Carl Zeiss GOM Metrology GmbH, Braunschweig, Germany) was used to record the subsidence and tilt of the implant relative to the bone and the deformation and micromotion of the bone relative to the embedding. The optical measurements were captured at a frequency of 30 Hz. Tracking points with a diameter of 0.8 mm were affixed to the embedding, bone, implant, and actuator, which facilitated the correction of rigid body motion in relation to the fixed embedding. Point clouds on the bone were placed in zone 5 of Denard et al. bone resorption classification as bone resorptions were clinically most present in this bone region [9]. The system’s dual-camera setup enabled spatial point cloud tracking with an average deviation of 4.9 ± 3.8 µm. Bone deformation was measured on the cortical superficial bone. The differentiation between the relative motion of the implant and the bone was accomplished by assigning different coordinate systems to each component within the optical tracking system.

Cyclic outcome variables (Figure 4A) retrieved from recorded images were compared either with the time-zero reference state (total bone deformation) or assessed during one load hysteresis applied (micromotion). Cyclic outcome variables regarding implant stability (Figure 4C) included implant tilt (α_imlant_) and subsidence (s_implant_) at the end of each loading block (220 N, 520 N, and 820 N). The measured bone deformation at the cortical surface during the final load cycle (final hysteresis width (HW)) between valley and peak loading offered insights into dynamic proximal bone loading (Bone micromotion − s_BoneHW_). Total bone deformation was measured in the medial calcar cortical bone from the time-zero reference image to the end of each loading block (Total bone deformation − s_BoneTot_). Testing the native and humeral component implanted situation allowed for a comparison of the bone deformation parameters (s_BoneHW_ and s_BoneTot_). The data were analyzed using a commercial software package (Matlab version R2023a, MathWorks, Natick, MA, USA).

### 2.5. Statistical Analysis

Biomechanical testing outcome metrics were the dependent primary outcome variables. Filling ratio calculations were used as covariates in multivariable regression analyses. Statistical analysis was performed using commercial software (JMP, version 17, JMP Statistical Discovery LLC, Cary, NC, USA).

Intraclass correlation coefficients (ICC) were used to examine the accuracy of the humeral canal fill ratios using a pre- to postoperative comparison. The analysis included a two-way random effects analysis for single measures, and reliability was applied in the context of consistency of a single measure and a single rater. ICCs greater than 0.75 were considered excellent, ICCs of 0.40 to 0.75 indicated moderate reliability, and ICCs of less than 0.40 indicated poor reliability [32,33].

Statistical analyses included one-way analysis of variance (ANOVA) with a Holm–Sidak post hoc test conducted for significant pairwise analysis of the primary outcome variables. A significance level of *p* ≤ 0.05 was established. The observed post hoc average power value of all one-way ANOVA tests exceeded the desired power level of 0.8, concluding that the sample size was sufficient. No prior sample size calculation was performed, as no matching mean and standard deviation values were found for our outcome variables and methods. The Shapiro–Wilks and Brown–Forsythe tests confirmed that each dataset represented a normal distribution and equal variance. A non-parametric test (Kruskal–Wallis) was used for datasets that failed these tests. For Kruskal–Wallis tests that found significance, Dunn’s post hoc tests including Bonferroni correction were conducted to further analyze the differences.

## 3. Results

Postoperative X-ray adjusted calculation of the filling ratios demonstrated improved reliability in preoperatively virtual positioned implants for the 3D VFR with an excellent ICC compared to moderate ICCs when using two-dimensional measures (Table 1).

The groupwise comparison of the canal fill measures resulted in significant differences when using the VFR for both groups and pre- and post-operative measures, while the two-dimensional measures showed a significant difference for the postoperative 2D Diaphysis measure only (Table 2). Specimen distribution (age and gender) did not have an effect on the filling ratio calculation or the bone density. No statistically significant differences were found in bone density and preoperative 2D measures (Table 2).

### 3.1. Primary Stability

**Figure 5:**
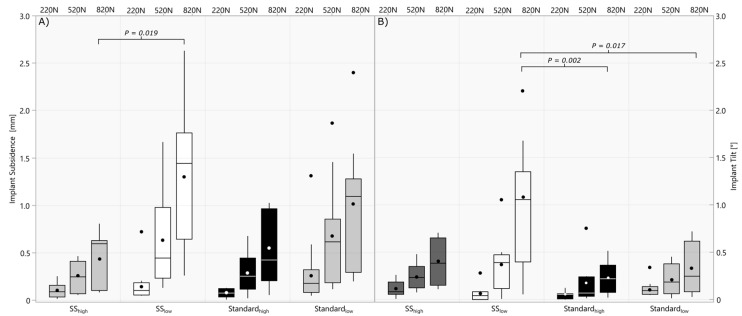
Boxplot of implant subsidence (**A**) and tilt (**B**) at the end of each cyclic loading block (220 N, 520 N, and 820 N) comparing short and standard stem implants, respectively, implanted with high and low filling ratios.

Lower canal fill ratios resulted in significantly higher variability between short and standard stems regarding implant tilt (820 N: *p* = 0.030) and subsidence (220 N: *p* = 0.046, 520 N: *p* = 0.007 and 820 N: *p* = 0.005). Among the short stems, implant subsidence was increased in the low filling ratio group compared to the high filling ratio group in the 820 N block (Figure 5A). The short stems in the low filling ratio group also showed significantly increased implant tilt at 820 N loading compared to standard stemmed implants with a low and high filling ratio (Figure 5B).

### 3.2. Bone Loading

**Figure 6:**
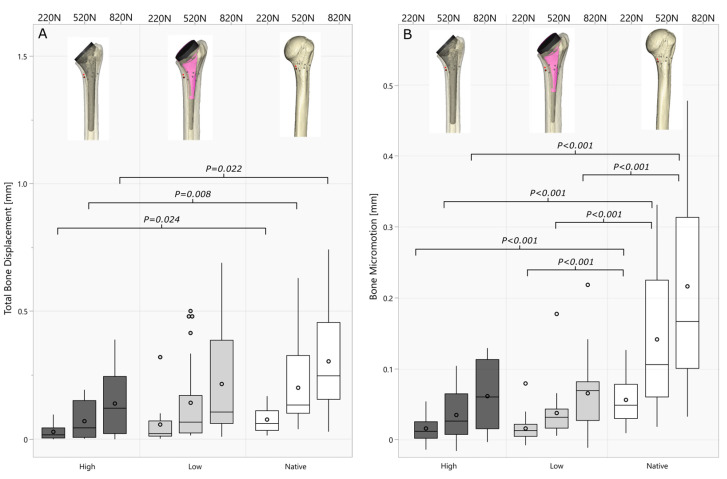
Boxplots of total bone deformation (**A**) and bone micromotion (**B**) for each cyclic loading block (220 N, 520 N, and 820 N) comparing low- and high filling ratios to the biomechanical behavior of the native bone.

No statistical differences in the bone loading variables were found between the short and standard stems with low or high filling ratios (*p* > 0.179), wherefore overall low and high filling ratio groups were compared including standard and short stems (Figure 6). Canal fill ratios across the groups (Range 0.54–0.97) significantly correlated with bone micromotion (220 N: r = 0.55 *p* < 0.001; 520 N: r = 0.52 *p* = 0.032) at lower load levels. Higher filling ratios resulted in significantly lower total bone deformation in the medial calcar area compared to the native bone (Figure 6A), while the total deformation in the lower filling ratio groups did not differ significantly (220 N: *p* = 0.374 520 N: *p* = 0.211; 820 N: *p* = 0.177). Testing of the native bone showed significantly increased bone micromotion compared to both lower and higher filling ratio groups (Figure 6B).

## 4. Discussion

The most important finding of this study was that preoperatively plannable volumetric canal filling ratios have significant effects on the biomechanical behavior of humeral components at the implant–bone interface. Higher humeral canal fill ratios reduced the implant-to-bone loading in the medial calcar bone region compared to the native bone. Lower canal fill ratios approximated the native bone deformations, while short stem implants with canal fill ratios < 0.72 demonstrated a higher risk for implant tilt and subsidence with biomechanical testing. A reliable and accurate method to calculate the preoperative filling ratio was developed explicitly for short and standard stem implants and validated with post-operative X-rays. This 2D to 3D registration was previously shown to allow accurate prediction of stress shielding based on a VFR in a retrospective cohort [14,16]. On the other hand, low filling ratios in RSA were shown to result in increased implant subsidence and tilt [12]. Experimental primary stability and bone deformation data of humeral RSA components for correlation analyses with the humeral canal fill ratio help to understand differing load transfer patterns and the deviations from the native bone loading.

Several studies have demonstrated a correlation between stem length or diameter and higher rates of proximal humerus stress shielding [18,34,35,36,37]. However, two-dimensional filling ratios calculated on plain radiographs can be affected by rotation, irregular geometry of the bony anatomy of the humerus, and the geometry of noncylindrical stems, resulting in only moderate accuracy [15]. Therefore, a more robust 3D measurement of the canal volume was used and validated in this work for filling ratio calculation. The application in preoperative CT scans from a standard clinical CT device ensured that the method is universally applicable in a preoperative planning process. The validation of the preoperative calculation using the postoperative position of the implant showed excellent reliability when using 3D models. Increased VFR ICCs (ICC = 0.835) compared to 2D measures (metaphyseal ICC = 0.569 and diaphyseal ICC = 0.495) demonstrate improved preoperative reliability and resulted in an improved separability between all low and high filling ratios and implant types while the 2D measurement only worked for short stem postoperative diaphyseal filling ratios (Table 2). A recent clinical retrospective study using 3D models and 2D postoperative registration in anatomic TSA showed good predictability of proximal humeral stress shielding based on volumetric metaphyseal and diaphyseal filling ratios [16]. The registration of preoperative CT data and postoperative X-rays benefitted our study as the canal fill ratios significantly affected the implant–bone loading and biomechanical device behavior. The increased reversible bone deformation suggests that stimulation can be maintained through the medial calcar region (zones 4 and 5) [9] when using lower filling ratios, which may reduce stress shielding caused bone resorption in this area, as observed clinically with an inlay or cortical rim supporting design [38,39]. Intended osseointegration at the bone–implant interface is closely associated with the mechanical environment of the implant and respective micromotions. Differently maintained mechanical bone loads have been reported to significantly affect bone restoration, especially during the proliferative phase of bone healing [40,41,42]. To reduce bone resorptions after the healing phase, bone micromotions mimicking the native load absorption pattern are desirable. In our study, the bone deformation measured in the medial calcar area was significantly shielded from load when comparing native and implantation test results. This correlates with clinical findings, as any metal implantation somehow stress shields the bone, even stemless designs result in medial calcar stress shielding [38,39,43,44]. In clinical studies, reduced bone resorption in lower filling ratios correlates with our findings where bone micromotions significantly correlate with the canal fill ratios. A reduction in bone loading significantly decreased the bone micromotion in high canal fill ratios (FR > 0.72) compared to the native bone, which may correlate with the severity of bone resorptions in the clinical setting.

During the application of the postoperatively relevant load levels (220 N, 520 N), the primary stability of lower filling ratios did not differ regarding stem type and filling ratio, similarly as shown in a recent biomechanical study in artificial bone [23]. However, lower filling ratios in combination with short stem implants were more prone to implant tilt and subsidence in increased (post-rehabilitation 820 N) loading. While higher filling ratios and standard stem implants withstood the 820 N load, higher loads during the rehabilitation protocol in lower canal fill ratios may cause earlier migration and tilt that may prevent bone ingrowth [27,45,46]. A recent biomechanical study investigated isolated stem stability and demonstrated significantly increased construct stiffness in +2 mm diameter increased short stems. The increased implant stability in higher filling ratios influenced the loading of the bone due to a more distally shifted implant-to-bone load transfer. Varus/valgus tilt, subsidence, different implant positions in the cancellous bed, and implant design and coatings significantly affect the primary humeral bone stresses [13,21,47,48,49]. Particularly the implant design used in this study, the flushlay design using a cup in the cancellous bed, contributes significantly to the primary stability and load transfer in the proximal humerus [50]. Therefore, the planning and inclusion of the cup size below the resection plane helped to determine the true volumetric filling ratio relevant to finding the trade-off between stress shielding and primary stability aiming for a ratio of 0.72, while still allowing adaption of the filling ratio. The adaption of implant sizes could help to gain a higher primary stability in poor bone densities where a final size prediction with the inclusion of bone density variables may pay off to patient-specifically find the most adequate implant sizes. Especially the volumetric canal fill ratio is more robust when adapting stem sizes, as the full construct humeral component is considered, compared to only two specific planes in 2D methods. However, the effects of differing implant designs on primary stability and bone adaptions during rehabilitation can be affected by other biological factors that influence bone formation or resorption [34,35,51].

While the effects of the preoperative evaluable bone density on biomechanical implant behavior have already been demonstrated, [17,24] this study showed significant effects of preoperatively calculated volumetric canal fill ratios on the primary stability and implant–bone loading. Both may influence primary implant stability after shoulder arthroplasty surgery, wherefore we controlled for an equal bone density distribution in the groups to reduce the impact of variable specimen age and gender between the groups and focus on the investigation of differing canal fill ratios. The impact of in vivo biologics such as the effects of bone ingrowth (secondary fixation) and stress shielding cannot be reproduced biomechanically, but the comparative findings of a native and implantation test setup help to understand potential causes for stress shielding and implant subsidence. Preoperative canal fill calculation allows to accurately determine the intended stem size to improve the planning process between the risk of stress shielding and limited implant stability.

There are some limitations to the current study. Bony adaptions which affect secondary stability in clinical applications, cannot be investigated in cadaveric biomechanical testing. Therefore, this study’s stability and bone loading results may behave differently in an in vivo setting over a more extended follow-up period. The effect of different implant designs, coatings, varying abduction angles as well as the micromotions in cancellous bone to promote bone ingrowth is a pertinent question beyond the scope of this study. The findings of this work may differ for onlay or inlay humeral component designs. The comparison of the combined groups of short and standard stems in the bone loading investigations can be improved to find bone loading differences between the stem length in specific filling ratios, maybe in an FE analysis. The usage of a short and standard stemmed implant using the same metaphyseal design allowed for the comparison to the native bone when applying low and high filling ratios. Volumetric canal fill ratio calculations were postoperatively verified; however, other deviations to preoperative planning (varus/valgus) may affect bone loading and subsidence patterns, which should be investigated accordingly. Additionally, the volume ratio only provides information on the implant and canal sizes without considering the influences associated with differing shapes and anatomies which may have an additional impact on the primary stability. An axial compression load vector was applied at a fixed angle to simulate the compressive loading of the humeral component. The test setup and method in this biomechanical study only roughly simulate the in vivo loading, and the implant may clinically behave differently. However, the findings using these implants showed the effects of stem and cup sizing on primary stability in a time-zero setting to show different load transfer patterns in an experimental biomechanical study. To overcome these limitations, the application of the volumetric filling ratio in preoperative planning should be studied in a prospective clinical setting. This approach may provide important information when comparing the biomechanical behavior of future stemless humeral RSA components to stemmed implants.

## 5. Conclusions

Both short and standard-length stem RSA humeral components implanted with a low canal filling ratio maintain dynamic bone loading in the medial calcar of the humerus similar to the native bone tested in this loading setup. However, the implantation of shorter stems with a lower filling ratio increased the risk of time-zero implant tilt and subsidence. In contrast, higher filling ratios or standard stems implanted with low or high filling ratios demonstrated higher primary stability, especially in higher daily peak loads (820 N). Volumetric preoperative canal fill calculations are more reliable than 2D calculations in planar radiographs.

## Figures and Tables

**Table 1:** Intraclass correlation coefficients (ICC) and confidence intervals (CI) assessing the consistency of the preoperative and postoperative canal fill calculation.

Canal Fill Ratio	ICC * (CI **)	*p*-Value
3D VFR ^#^	0.835 (0.710–0.910)	<0.001
2D Metaphysis FR ^$^	0.569 (0.316–0.746)	<0.001
2D Diaphysis FR ^$^	0.495 (0.220–0.697)	<0.001

* ICC intra class correlation coefficient; ** CI confidence interval; ^#^ VFR volumetric filling ratio; ^$^ FR filling ratio.

**Table 2:** Mean values with standard deviations including statistical analysis of the density variables (BMD Bone Mineral Density; BV/TV Bone Volume/Total Volume) for specimens assigned to the standard or short stem groups with low and high filling ratios, respectively.

Imaging Parameter	Standard _Low_	Standard _High_	*p*-Value	SS^}^ _Low_	SS^}^ _High_	*p*-Value	*p*-Value Standard _Low_ vs. SS^}^ _Low_	*p*-Value Standard _High_ vs. SS^}^ _High_
Age [years]	66± 5	66 ± 5	0.999	66 ± 3	66 ± 4	0.999	0.999	0.999
Number Females	4	5	-	3	4	-	-	-
Epi. Cyl. BMD ** [mgHA/cm^3^]	305 ± 39	322 ± 49	0.789	300 ± 49	287 ± 25	0.903	0.991	0.263
Epiphysis Cylinder BV/TV ~	0.31 ± 0.04	0.33 ±0.04	0.542	0.34 ± 0.07	0.31 ± 0.04	0.499	0.333	0.719
Metaphysis Cyl. BV/TV ~	0.21 ± 0.03	0.24 ± 0.03	0.393	0.23 ± 0.04	0.21 ± 0.04	0.742	0.662	0.468
Inf. Sup. BMD ** [mgHA/cm^3^]	356 ± 43	355 ± 38	0.999	335 ± 36	325 ± 36	0.929	0.698	0.368
3D VFR_PreOP_^#^	0.62 ± 0.06	0.77 ± 0.10	0.003 *	0.62 ± 0.07	0.80 ± 0.10	<0.001 *	0.999	0.814
3D VFR_PostOP_^#^	0.65 ± 0.08	0.77 ± 0.11	0.013 *	0.62 ± 0.05	0.82 ± 0.09	<0.001 *	0.925	0.690
2D Metaphysis FR_PreOP_^$^	0.62 ± 0.06	0.65 ± 0.05	0.610	0.66 ± 0.06	0.70 ± 0.05	0.404	0.557	0.357
2D Diaphysis FR_PreOP_^$^	0.50 ± 0.05	0.52 ± 0.06	0.882	0.49 ± 0.04	0.55 ± 0.05	0.085	0.975	0.570
2D Metaphysis FR_PostOP_^$^	0.61 ± 0.05	0.62 ± 0.05	0.991	0.64 ± 0.05	0.63 ± 0.0+	0.965	0.982	0.675
2D Diaphysis FR_PostOP_^$^	0.50 ± 0.07	0.52 ± 0.05	0.950	0.49 ± 0.05	0.57 ± 0.07	0.014 *	0.969	0.175

* statistical significance (*p* < 0.05); ** BMD Bone mineral density; ~ BV/TV bone volume per total volume; ^#^ VFR volumetric filling ratio; ^$^ FR filling ratio; ^}^ SS short stem.

## Data Availability

Dataset available on request from the authors.
